# Supplementary material for: Electrostatic complementarity at the interface drives transient protein-protein interactions
Source: Sci Rep. 2023 Jun 23;13:10207. doi: 10.1038/s41598-023-37130-z (PMC10290103; doi:10.1038/s41598-023-37130-z)
Supplement: Supplementary file 1 — Supplementary Information. [file 41598_2023_37130_MOESM1_ESM.pdf]

Supporting Materials - Electrostatic complementarity at the interface drives transient protein-protein interactions

Greta Grassmann,[1,2] Lorenzo Di Rienzo,[2] Giorgio Gosti,[2,3] Marco Leonetti,[2,3]
Giancarlo Ruocco,[2,4] Mattia Miotto,[2,5,6] and Edoardo Milanetti[4,2,5,6]

[1]*Department of Biochemical Sciences "Alessandro Rossi Fanelli", Sapienza University of Rome, P.Le A. Moro 5, 00185, Rome, Italy*
[2]*Center for Life Nano & Neuro Science, Istituto Italiano di Tecnologia, Viale Regina Elena 291, 00161, Rome, Italy*
[3]*Soft and Living Matter Laboratory, Institute of Nanotechnology, Consiglio Nazionale delle Ricerche, 00185, Rome, Italy*
[4]*Department of Physics, Sapienza University, Piazzale Aldo Moro 5, 00185, Rome, Italy*
[5]*The authors contributed equally to the present work.*
[6]*Corresponding authors:*
mattia.miotto@roma1.infn.it
edoardo.milanetti@uniroma1.it

I. ADDITIONAL FIGURES AND TABLES

**Complex**	**Dimer**	**Structure**
1bd9	SBR-hom	SS
1bht	nIBR-hom	SS
1a4r	SBR-hom	HH
1c3i	SBR-hom	HH
1azv	SBR-hom	SS
1ekf	SBR-hom	SS
1d6n	nIBR-hom	HH
1deh	SBR-hom	HH
1ckg	SBR-hom	HH
1f05	SBR-hom	HH
1f3h	IBR-hom	HH
1itq	nIBR-hom	HH
1il0	IBR-hom	HH
1i3k	IBR-hom	HH
1jcq	nIBR-het	HH
1j1b	nIBR-het	HH
1jt3	nIBR-hom	SS
1iv5	SBR-hom	HH
1j2e	IBR-hom	SS
1k6m	nIBR-hom	HH
1l8l	SBR-hom	HH
1kfu	nIBR-het	HH
1k3y	SBR-hom	HH
1juo	SBR-hom	HH
1k9k	SBR-hom	HH
1m6h	SBR-hom	HH
1m4r	SBR-hom	HH
1nbq	SBR-hom	SS
1p4r	nIBR-hom	HH
1pgt	SBR-hom	HH
1pe0	IBR-hom	HH
1psr	nIBR-hom	HH
1qha	nIBR-hom	HH
1qin	SBR-hom	HH
1qr2	nIBR-hom	HH
1t09	SBR-hom	HH
1szb	SBR-hom	SS
1qpf	SBR-hom	SS
1tgz	nIBR-het	SH
1t4e	SBR-hom	HH
1tjc	nIBR-hom	HH
1vec	nIBR-hom	HH
1u4l	SBR-hom	SH
1wrk	nIBR-hom	HH
1wsr	SBR-hom	SS
1xw5	IBR-hom	HH
1ypq	SBR-hom	SS
1yb5	IBR-hom	HH
1y7v	nIBR-hom	SH
1yfk	IBR-hom	HH

**Complex**	**Dimer**	**Structure**
1z68	SBR-hom	SS
1zn8	IBR-hom	HH
1zq9	nIBR-hom	HH
1zuo	nIBR-hom	HH
1yzx	nIBR-hom	HH
2avd	IBR-hom	HH
2ask	IBR-hom	SS
2ath	nIBR-hom	HH
2a1j	nIBR-het	HH
2cb5	nIBR-hom	HH
2dc3	nIBR-het	HH
2egd	nIBR-het	HH
2eav	SBR-hom	HH
2gh5	nIBR-hom	HH
2g76	nIBR-hom	HH
2grn	nIBR-het	HH
2gk2	SBR-hom	SS
2h2n	IBR-hom	SS
2h4x	IBR-hom	HH
2ha8	nIBR-het	HH
2hp4	nIBR-hom	SS
2hdj	SBR-hom	HH
2he0	IBR-hom	HH
2hqx	nIBR-hom	SS
2i7d	IBR-hom	HH
2i99	IBR-hom	HH
2hth	nIBR-het	SS
2ofx	IBR-hom	HH
2o53	IBR-hom	HH
2o06	SBR-hom	HH
2pla	IBR-hom	HH
2pn7	nIBR-het	SS
2qjf	SBR-hom	HH
2qpp	nIBR-het	HH
2r83	nIBR-hom	SS
2z5d	nIBR-het	HH
2z5f	nIBR-hom	HH
3bm4	nIBR-hom	SS
3bs9	IBR-hom	SS
3c3s	SBR-hom	HH
3ee2	SBR-hom	HH
3cnk	SBR-hom	SS
3g4e	nIBR-hom	SS
3g3d	IBR-hom	HH
3f3s	nIBR-hom	HH
3f8g	IBR-hom	SS
3ga1	IBR-hom	HH
3gov	nIBR-het	SS
3h30	nIBR-hom	HH
3gix	SBR-hom	HH

**Complex**	**Dimer**	**Structure**
3h53	SBR-hom	HH
3hkv	nIBR-hom	SS
3hju	nIBR-het	HH
3jus	nIBR-hom	HH
3hup	SBR-hom	SS
3kv6	nIBR-hom	HH
3lf5	nIBR-hom	HH
3lgd	nIBR-hom	HH
3lxb	SBR-hom	HH
3lhr	IBR-hom	HH
3mqm	nIBR-hom	HH
3mdg	IBR-hom	SS
3mtr	nIBR-het	SS
3mgm	IBR-hom	HH
3n8e	SBR-hom	SS
3mzg	nIBR-het	SH
3nl7	nIBR-het	HH
3ooy	nIBR-hom	HH
3osk	SBR-hom	SS
3o2s	nIBR-het	HH
3pdj	nIBR-het	HH
3q18	IBR-hom	HH
3smj	nIBR-hom	SS
3twq	nIBR-hom	HH
3v8c	IBR-hom	SS
3up1	nIBR-hom	SS
3umz	nIBR-hom	SS
3vpm	nIBR-hom	HH
4dkc	SBR-hom	HH
4f5y	IBR-hom	HH
4en4	SBR-hom	HH
4g03	nIBR-hom	HH
4hfg	IBR-hom	HH
4hw5	nIBR-hom	HH
4idn	nIBR-het	HH
4gr7	IBR-hom	SS
4inc	SBR-hom	HH
4iy4	IBR-hom	HH
4onl	nIBR-het	HH
4oo4	IBR-hom	HH
4p2y	nIBR-het	SH
4ors	SBR-hom	SS
4oz0	nIBR-hom	HH
4pzg	SBR-hom	HH
4rca	nIBR-het	SS
4r14	SBR-hom	SS
4run	nIBR-het	SS
4w5v	nIBR-het	SH
4uc4	SBR-hom	SS
4wii	nIBR-het	HH

**Complex**	**Class**	**Structure**
4x6x	IBR-hom	HH
4y2h	nIBR-hom	SH
4zcb	nIBR-hom	SS
4z18	nIBR-het	SS
4za0	nIBR-hom	HH
4yep	SBR-hom	SS
4z9o	nIBR-het	HS
4zmv	SBR-hom	SS
4zte	SBR-hom	SS
5btq	SBR-hom	HH
5b0h	nIBR-hom	SS
5d7p	SBR-hom	HH
5f1a	SBR-hom	HH
5duq	nIBR-hom	HH
5f9s	nIBR-hom	HH
5h9q	IBR-hom	SS
5hpg	IBR-hom	SS
5j8e	SBR-hom	HH
5jg8	SBR-hom	HH
5l73	nIBR-het	SS
5lvr	nIBR-het	HH
5mgr	nIBR-hom	SS
5m4g	nIBR-hom	HH
5mol	nIBR-hom	SS
5lxf	SBR-hom	HH
5ohh	SBR-hom	SS
5o10	nIBR-hom	HH
5t3v	IBR-hom	HH
5u0i	IBR-hom	HH
5vr6	SBR-hom	HH
5vbr	SBR-hom	HH
5uq2	nIBR-het	HH
5vxa	IBR-hom	HH
5x67	IBR-hom	HH
5wi2	IBR-hom	SS
6bml	nIBR-het	HH
6bqu	IBR-hom	HH
5y15	nIBR-het	HH
6cic	SBR-hom	HH
6esy	IBR-hom	HH
6fb4	SBR-hom	SS
6dvr	nIBR-hom	SS
6g9z	IBR-hom	HH
6g6s	SBR-hom	HH
6gfb	SBR-hom	HH
6fvz	SBR-hom	HH
6fge	nIBR-het	SH
6giu	SBR-hom	HH
6gzm	SBR-hom	HH

TABLE I: **'Human' dataset.** For each of the 199 considered complexes the name of the pdb, the classification of the dimer and the classification of the secondary structure are reported respectively in the *Complex*, *Dimer* and *Structure* columns.

Complex	B_a	Complex	B_a	Complex	B_a
1jtd	-10.57	1y34	-9.28	2uuy	-8.25
1bvn	-11.05	1wr1	-4.83	2ptt	-5.4
1j7d	-5.7	1wrd	-3.39	2uyz	-7.09
1emv	-13.62	1ugh	-10.92	2rnr	-6.82
1grn	-6.41	1y33	-8.77	2omw	-5.0
1jiw	-11.4	1y4a	-8.92	2qc1	-11.0
1e96	-5.57	1y3d	-9.25	2wp3	-6.1
1j7v	-10.46	1yx6	-4.14	2v9t	-8.09
1ay7	-6.0	1zgu	-4.01	2vlq	-10.64
1lp1	-5.7	1y4d	-8.92	2wwk	-5.89
1lzw	-6.48	1y3c	-10.6	2wo3	-5.64
1l8c	-8.15	1y6k	-9.0	2wy8	-6.44
1m10	-8.24	1yx5	-3.46	3bh6	-7.02
1kac	-7.83	1y48	-8.77	2z58	-7.8
1lx5	-8.92	2a9k	-7.22	2vlp	-12.27
1lw6	-11.7	1zvy	-10.0	3knb	-6.03
1ry7	-6.64	1zv5	-8.0	3ixe	-5.64
1ta3	-8.05	2b12	-4.7	3kuc	-6.35
1t0p	-4.6	2a78	-7.22	3kud	-5.77
1sq0	-7.52	2hrk	-8.05	3f1p	-3.92
1op9	-9.15	2jti	-6.23	3hct	-5.83
1ri8	-8.54	2k2s	-7.28	3k1r	-9.0
1r8u	-7.89	2hev	-7.21	3bn3	-4.7
1te1	-8.47	2jy6	-4.7	3kw5	-6.41
1tm5	-10.77	2jt4	-4.4	3e1z	-10.44
1tdq	-7.92	2hth	-3.98	3fpu	-9.92
1tm1	-11.52	2f4m	-7.19	3fju	-8.8
1tm7	-11.48	2fuh	-3.52	3ncc	-0.67
1u0s	-6.64	2k6d	-3.77	3n0p	-2.29
1tm3	-10.72	2k3s	-5.57	3me2	-10.17
1to1	-9.34	2k5b	-5.4	3m18	-9.74
1tlh	-3.52	2k8c	-2.74	3ncb	-1.03
1tm4	-9.89	2k8b	-2.74	3nvn	-8.03
1tba	-9.0	2k79	-3.17	3n06	-2.51
1vet	-7.89	2k7a	-3.17	3mzg	-2.3
1wq1	-4.77	2o3b	-11.49	3qq8	-5.82
1y1k	-9.52	2knb	-4.96	3tnf	-11.0
1xg2	-8.3	2nqd	-10.41	3ona	-6.17
1wqj	-6.06	2omu	-9.22	3t04	-7.28
1u5s	-2.52	2ka4	-7.24	3qc8	-4.95
1y3b	-9.51	2omx	-6.92	3uyo	-5.17

TABLE II: **'Affinity' dataset.** For each of the 123 considered complexes the name of the pdb and the B_a are reported.

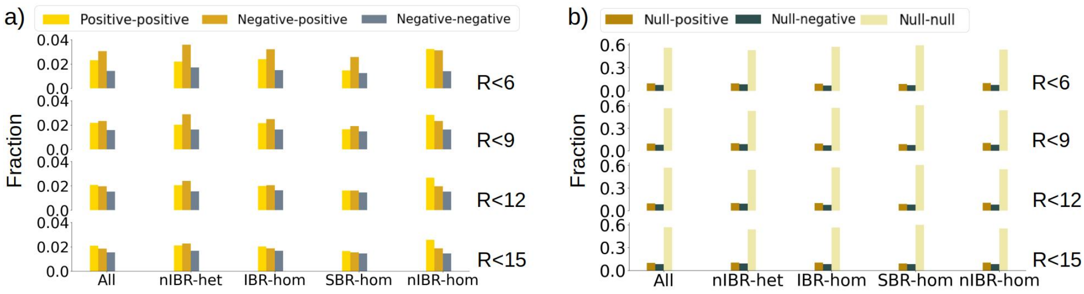

FIG. 1: **Charge properties of the dataset. a)** On each interacting surface residue a sphere of radius R is built, and the number of interacting residues on the partners surface included in the sphere is counted. The bar plot shows, for increasing values of R (as reported by the labels on the right) and for both the whole dataset and each of the four classes, the fraction of positively or negatively charged residues that can be found close to positive residues, respectively in yellow or ochre. In grey, the fraction of negative residues closed to a negative amino acid. **b)** For increasing values of R (as reported by the labels on the right) and for both the whole dataset and each of the four classes, the fraction of positively, negatively or null residues that can be found close to non charged residues, respectively in brown, beige and grey.

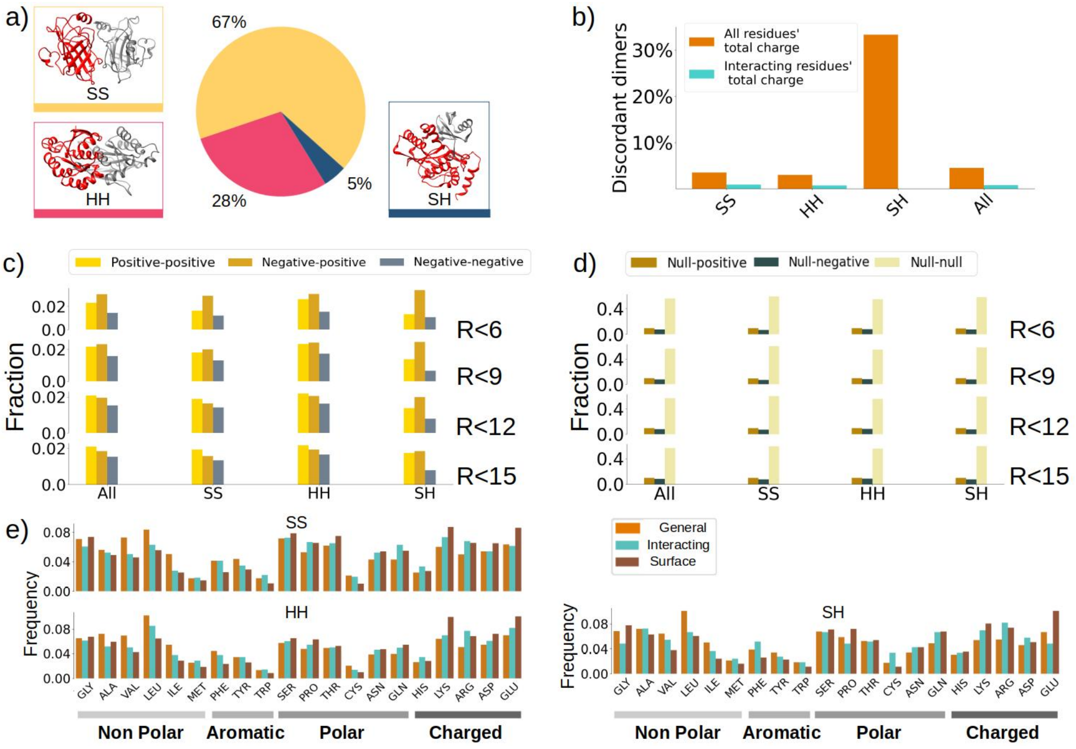

FIG. 2: **Structural classification of the 'Human' dataset, amino acid composition and charge properties of the classes.** **a)** The complexes in the dataset are divided into SS, HH and SH. The colored boxes report an example for each category. The same colors are used to indicate in the pie chart each class abundance in the dataset. **b)** For each protein, the sum of the charges of all its residues and only the interacting residues on the surface is computed. For each complex, these total and interacting charges from the two interacting partners are multiplied. The bar plot shows, for the whole dataset and each class, the percentage of complexes whose total (in orange) and interacting (in blue) products are negative. **c)** On each interacting surface residue a sphere of radius R is built, and the number of interacting residues on the partners surface included in the sphere is counted. The bar plot shows, for increasing values of R (as reported by the labels on the right) and for both the whole dataset and each of the three classes, the fraction of positively or negatively charged residues that can be found close to positive residues, respectively in yellow or ochre. In grey, the fraction of negative residues closed to a negative amino acid. **d)** For increasing values of R (as reported by the labels on the right) and for both the whole dataset and each of the three classes, the fraction of positively, negatively or null residues that can be found close to non charged residues, respectively in brown, beige and grey. **e)** The relative abundances of each of the twenty natural amino acids considering all the residues (orange), only the interacting ones (in green), and only the solvent-exposed residues (brown) are shown. The results are divided into the three classes.

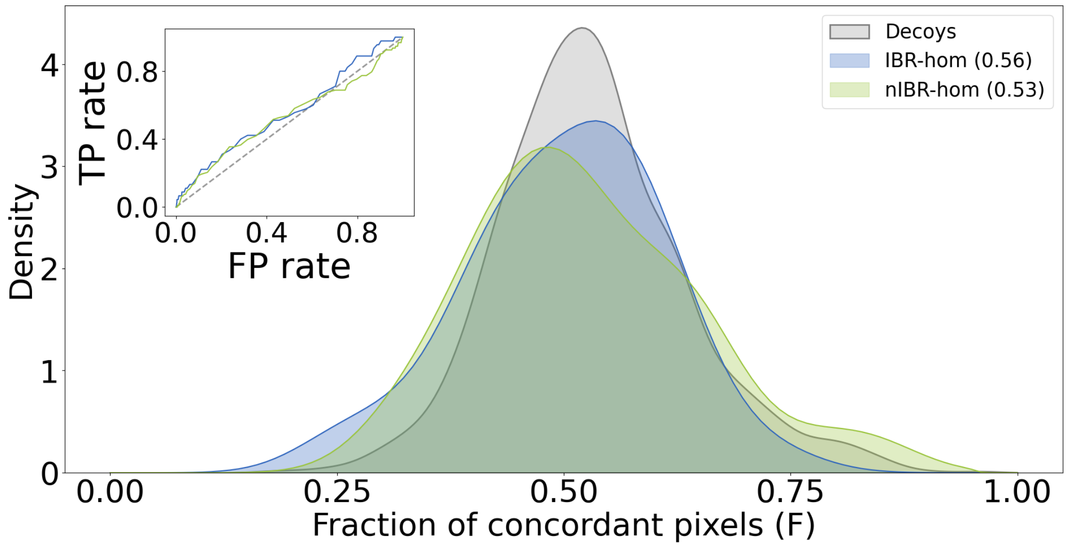

FIG. 3: **Electrostatic complementarity contribution in protein-protein complexes.** Distributions of the F values of the interacting patches in complexes from the IBR-hom (blue) and nIBR-hom (green) classes. In the insert the corresponding ROC curves.

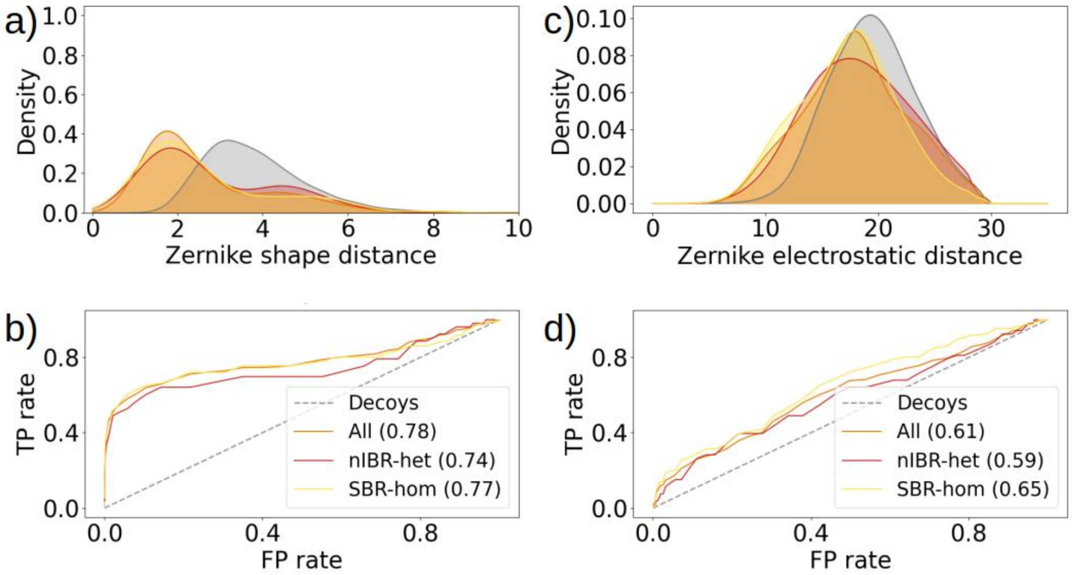

FIG. 4: **2D Zernike polynomials to compare surfaces regions.** **a)** Distributions of the distances between the Zernike vectors describing the molecular surface of nIBR-het and SBR-hom interacting (red and yellow respectively) and random (grey) patches in the Human dataset. The distribution of all the patches in the dataset is shown in orange. **b)** ROC curves of the distributions in a) and corresponding AUC (in the legend) computed against the random distribution. **c)** For each patch the distance between the Zernike vectors describing the electrostatic potential surface in that region is computed. Then the same analysis and classification as in a) is performed. **d)** ROC curves of the distributions in c) and corresponding AUC (in the legend) computed against the random distribution.

II. PREDICTION TESTING

To test the observed anti-correlation between binding affinity and electrostatic complementarity we selected a third dataset, that we call 'Variants' dataset. This dataset includes five SARS-CoV-2 variants of concerns (VOCs) (alpha, beta, gamma, delta and omicron) with known dissociation constant (K_d) [1].
The 'Variants' dataset was obtained starting from the experimentally resolved structure of the wild-type (WT) spike protein bound to Angiotensin-Converting Enzyme 2 (ACE2) (pdb id: 6M0J). Since not all the VOCs have an available experimental structure, WT was subjected to computational mutagenesis using the dedicated tool provided in the PyMol software [2]. We selected the ACE2 residues from 19 to 615 in complex with spike residues from 333 to 526, and we only considered the mutations in the spike Receptor Binding Domain (RBD), including residues from 319 to 541, because those are the interacting regions [3, 4]. For each complex, we performed with Gromacs 2020.6 [5] a 100-ns-long molecular dynamics simulation and extracted configurations of the system every 1 ns. For this dataset, to reduce the computational time, the centers of all the frames' interacting regions were defined using the starting structure of the spike protein original version. We super-positioned each structure with the original spike protein, and selected the points closest to the binding region on this original version. To increase the volume of our data despite the low number of complexes, for each frame we defined N pairs of interacting patches, where N corresponds to the 5% of the points forming the surface mesh included in that interacting region. To avoid redundancy in the analysis we defined the patches with a radius of 6 Å. Since for this dataset we only performed the Zernike-based complementarity evaluation, this radius value was already shown to result in the highest efficiency [6]. The so-defined interacting patches include the points of the electrostatic surfaces that are projected in the electrostatic matrices. The electrostatic surfaces and the electrostatic matrices were instead obtained as described in the Methods in the main text.
We then applied the Zernike formalism to measure the complementarity between ACE2 and each variants for all the simulation frames. Table III shows for each variant the experimental K_d and the shape (Z_s column) and electrostatic (Z_{el} column) complementarities measured in terms of Euclidean distances between the Zernike descriptors, as discussed in the main text. As expected, complexes with lower K_d (higher binding stability) have higher shape complementarity (smaller Z_s values) because the role of the Lennard-Jones potential predominates. On the other hand, complexes with higher K_d (lower binding affinity) tend to have a higher electrostatic complementarity (smaller Z_{el} values) because they exploit Coulombic complementarity to acquire specificity. This would seem to confirm that electrostatic complementarity has greater role in less stable complexes.

**Variant**	K_D	Z_s	Z_{el}
Alpha	5.40	2.97	17.88
Gamma	11.00	2.96	17.96
Beta	13.83	2.96	18.14
Delta	25.07	3.01	17.40
Omicron	31.40	3.05	16.87

TABLE III: **Dissociation constant and shape and electrostatic complementarity for five SARS-CoV-2 variants.** List of the SARS-CoV-2 variants considered in this study with their K_d as measured by Han, P. *et al* [1] in nM, and the shape (Z_s column) and electrostatic (Z_{el} column) complementarities measured in terms of Euclidean distances between the Zernike descriptors.

[1] Pengcheng Han, Linjie Li, Sheng Liu, Qisheng Wang, Di Zhang, Zepeng Xu, Pu Han, Xiaomei Li, Qi Peng, Chao Su, et al. Receptor binding and complex structures of human ace2 to spike rbd from omicron and delta sars-cov-2. *Cell*, 185(4):630–640, 2022.

[2] Warren L DeLano et al. Pymol: An open-source molecular graphics tool. *CCP4 Newsl. Protein Crystallogr*, 40(1):82–92, 2002.

[3] Renhong Yan, Yuanyuan Zhang, Yaning Li, Lu Xia, Yingying Guo, and Qiang Zhou. Structural basis for the recognition of sars-cov-2 by full-length human ace2. *Science*, 367(6485):1444–1448, 2020.

[4] Gabriele Cerutti, Yicheng Guo, Tongqing Zhou, Jason Gorman, Myungjin Lee, Micah Rapp, Eswar R Reddem, Jian Yu, Fabiana Bahna, Jude Bimela, et al. Potent sars-cov-2 neutralizing antibodies directed against spike n-terminal domain target a single supersite. *Cell host & microbe*, 29(5):819–833, 2021.

[5] David Van Der Spoel, Erik Lindahl, Berk Hess, Gerrit Groenhof, Alan E Mark, and Herman JC Berendsen. Gromacs: fast, flexible, and free. *Journal of computational chemistry*, 26(16):1701–1718, 2005.

[6] Edoardo Milanetti, Mattia Miotto, Lorenzo Di Rienzo, Michele Monti, Giorgio Gosti, and Giancarlo Ruocco. 2d zernike polynomial expansion: Finding the protein-protein binding regions. *Computational and Structural Biotechnology Journal*, 19:29–36, 2021.
